# Investigation of first ray mobility during gait by kinematic fluoroscopic imaging-a novel method

**DOI:** 10.1186/1471-2474-13-14

**Published:** 2012-02-09

**Authors:** Heiner Martin, Ulf Bahlke, Albrecht Dietze, Volker Zschorlich, Klaus-Peter Schmitz, Thomas Mittlmeier

**Affiliations:** 1 Institute for Biomedical Engineering, University of Rostock, F. Barnewitz-Str. 4, D-18119 Rostock, Germany; 2 Dept. of Trauma and Reconstructive Surgery, University of Rostock, Schillingallee 35, D-18057 Rostock, Germany; 3 Institute for Sports Biomechanics and Sports Science, University of Rostock, Ulmenstraße 69, Building #2, D-18057 Rostock, Germany

## Abstract

**Background:**

It is often suggested that sagittal instability at the first tarso-metatarsal joint level is a primary factor for hallux valgus and that sagittal instability increases with the progression of the deformity. The assessment of the degree of vertical instability is usually made by clinical evaluation while any measurements mostly refer to a static assessment of medial ray mobility (i.e. the plantar/dorsal flexion in the sagittal plane). Testing methods currently available cannot attribute the degree of mobility to the corresponding anatomical joints making up the medial column of the foot. The aim of this study was to develop a technique which allows for a quantification of the in-vivo sagittal mobility of the joints of the medial foot column during the roll-over process under full weight bearing.

**Methods:**

Mobility of first ray bones was investigated by dynamic distortion-free fluoroscopy (25 frames/s) of 14 healthy volunteers and 8 patients with manifested clinical instability of the first ray. A CAD-based evaluation method allowed the determination of mobility and relative displacements and rotations of the first ray bones within the sagittal plane during the stance phase of gait.

**Results:**

Total flexion of the first ray was found to be 13.63 (SD 6.14) mm with the healthy volunteers and 13.06 (SD 8.01) mm with the patients (resolution: 0.245 mm/pixel). The dorsiflexion angle was 5.27 (SD 2.34) degrees in the healthy volunteers and increased to 5.56 (SD 3.37) degrees in the patients. Maximum rotations were found at the naviculo-cuneiform joints and least at the first tarso-metatarsal joint level in both groups.

**Conclusions:**

Dynamic fluoroscopic assessment has been shown to be a valuable tool for characterisation of the kinematics of the joints of the medial foot column during gait.

A significant difference in first ray flexion and angular rotation between the patients and healthy volunteers however could not be found.

## Background

First ray and first metatarsal are commonly used interchangeably when referring to the first metatarsal-cuneiform arch segment [1] despite the fact that first metatarsal corresponds to a single bony structure whereas the first metatarsal-cuneiform arch segment consists of the first metatarsal, the adjacent medial cuneiform and the navicular bone. Hypermobility of the first ray, mainly due to plantar arch and Lisfranc joint ligaments laxity, is assumed to predispose for hallux valgus in a certain subset of patients [1,2]. The fraction of these patients with manifested instability of the first ray has been reported to range between 10% [3] and 94% [4] of all patients with symptomatic hallux valgus deformity. With increasing degree of hallux valgus deformity and adduction of the first metatarsal, the first ray may become hypermobile and symptomatic [5,6]. Increased dorsal extension of the first metatarsal subsequently leads to an unloading of the first ray and a load shift to the lesser metatarsals during weight-bearing and may induce metatarsalgia [6-8]. Furthermore, while the medial arch of the foot lowers during weight-bearing, the first metatarsal axis orients more vertically and drives the first metatarsal into increased adduction promoting the hallux valgus deformity [1,6].

Excessive pronation is believed to occur during gait due to ligamentous laxity which may delay supination of the midtarsal and subtalar joints, decreases the rigidity of the foot during the terminal stance phase and adversely affects the push-off mechanics [7]. Despite the fact that three-dimensional motions occur at the medial foot column during weight-bearing [2,8] it appears that the major component of motion for the first metatarsal can be located in the sagittal plane [7].

The need for a quantification of vertical instability of the first ray has been postulated for decades [2,6-8]. Clinically the definition of hypermobility, even with the help of load-bearing radiography [5], is largely subjective [2]. Some authors have developed mechanical devices which allow for a static measurement of first ray mobility compared to the fixed metatarsal rays 2 to 5 [9-12]. The two most frequently applied devices possess similar diagnostic accuracy and yield an average normal dorsal mobility between 4.9 mm and 5.2 mm [13]. According to these methods, 8 mm of sagittal motion was regarded as the threshold of first ray hypermobility [13]. Unfortunately, the few studies employing dynamic gait examination methods revealed a higher scatter of normal first ray sagittal plane motion and did not show any significant association between the static measure of first ray mobility and dynamic first ray motion [7,14].

It is known from in-vitro experiments that there is a substantial difference between the corresponding motions of the specific joints of the first ray and the adjacent joints (i.e. the talo-navicular joint, the naviculo-cuneiform joint and the medial cuneiform-first metatarsal joint) [15]. The corresponding motions of the specific joints of the first ray and the adjacent joints are difficult to quantify by routine motion analysis techniques employing skin markers. The known problems associated with skin motion and the adjacent structures building the first ray limit the resolution and the possible anatomic discrimination without making use of invasive methods [16-18]. Hence, it was the aim of the present investigation to develop an in-vivo method of analysing first ray dorsi-/plantar flexion from distortion-free fluoroscopic image sequences of the foot rolling motion during gait of healthy and pathologically altered feet. In particular, it was thought that analysis of distortion-free fluoroscopic image sequences might clearly demonstrate differences of the dynamic medial cuneiform-first metatarsal joint motion, if any, between normal and hallux valgus patients with clinically manifested first ray instability.

## Methods

### Fluoroscopic imaging and gait analysis

**Figure 1:**
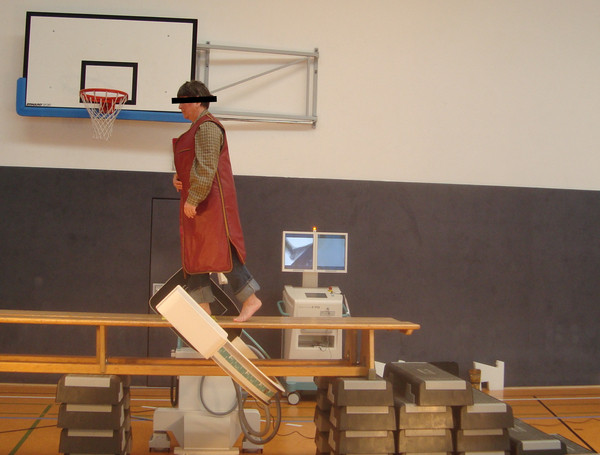
**Experimental set-up during data acquisition by dynamic fluoroscopic imaging**. The testing person is performing a complete roll-over process of the gait cycle within the measurement field of the flat panel detector which is turned to an oblique position.

Measurements were based on lateral view fluoroscopic image sequences from a single foot roll-over portion of the gait cycle detected by a mobile digital fluoroscopic imaging device (Ziehm Vision RFD, Ziehm Imaging GmbH, Nuremberg, Germany, Figure 1). The flat panel detector employs amorphous silicon photodiode TNT technology with a field size of 29.8 cm × 29.8 cm and a detector matrix of 1536 × 1536 pixels (data as supplied by manufacturer). Using the magnification option 1, an active field of 1024 × 1024 pixels with a local resolution of 0.245 pixel/mm was available. An important feature of this device is that it produces distortion-free images which permit in-plane image evaluation without correction. Another important feature is that pulsed scanning is used to produce the distortion-free images. Pulsed scanning reduces the amount of radiation exposure. Sample rate was 25 images per second.

The imaging device permitted a differentiated assessment of the relative motions of the several bones of the medial foot column with their articulations. Due to the two-dimensional projection images, the evaluation had to be restricted to the motions in the sagittal plane, only. The local ethical committee had reviewed and consented to the study protocol (A201073).

### Test method and tested persons

Twenty-two subjects, all of whom gave their written consent, participated in the study. Thus, a total of 44 feet were investigated. The control group consisted of fourteen symptom-free persons without clinically manifested foot problems. The experimental group consisted of eight patients who were scheduled for surgical treatment for hallux valgus deformity. All of the patients had clinically manifested instability of the first ray according to the assessment of two experienced orthopaedic surgeons not involved in these experiments. Determination of relevant, pathological first ray instability was determined by clinical testing using the drawer test of the first tarsometatarsal joint and observing the patients' discomfort during the procedure. The average age was 36 (SD 10, range 24-54) years for the healthy volunteers, and 45 (SD 16, range 15-66) years for the patients.

Fluoroscopic imaging was performed as shown in Figure 1. The bench walkway was elevated to accommodate the fixed dimensions of the C-arm and to allow lateral positioning of the imaging device. The fluoroscopic imaging field of view was marked on the walkway.

During measurements all tested persons wore circumferential radiation protection gowns including a thyroid gland protection lace leaving their lower legs and feet free. It was assumed that the aprons did not affect the normal gait of the persons. All persons involved into the data acquisition process were outside of the control area. In order to minimize radiation exposure, repetitive data acquisition was not employed. At the start of the measurement, the roll-over range of the first step was aligned in such a way that the foot was exactly within the imaging field of view. A complete foot roll-over cycle was documented from heel strike until lift-off of the toes.

The fluoroscopic image sequences showed the foot rolling-over two-dimensionally. The video was subdivided into frames of 40 ms intervals.

During the measurements, it was ensured that the left and the right foot were placed in such a way that the first ray bones were approximately the same distance from the x-ray source. Any further errors resulting from projection were neglected. Moreover, an equal scale for all images was assumed, as the zoom factor was not changed during the recordings. The image scale was calibrated by using a sphere with a known diameter of 40 mm. The sphere was placed into the center plane of the walking field and its diameter in pixels was measured.

Care was taken that the foot always moved parallel to the projection plane during the measurements. The test persons were asked to set their foot in question within the imaging range which was marked within the walkway. Moreover, they were asked to walk straight along the center line of the walkway.

### Evaluation

The image data were imported into a CAD system (Solid Works 2007, Dassault systèmes, Vélizy-Villacoublay, France) for the calculation of position and orientation of the bone projections. The evaluation continued with the manual drawing of the outline of the individual first ray bones. The manual segmentation procedure was used since the overlay of the bone contours did not allow the application of automatic segmentation algorithms. The following bones were analyzed with respect to first ray mobility (i.e. dorsi-/plantar flexion and rotations of the bones in the sagittal plane): first metatarsal, medial cuneiform, navicular and talus.

**Figure 2:**
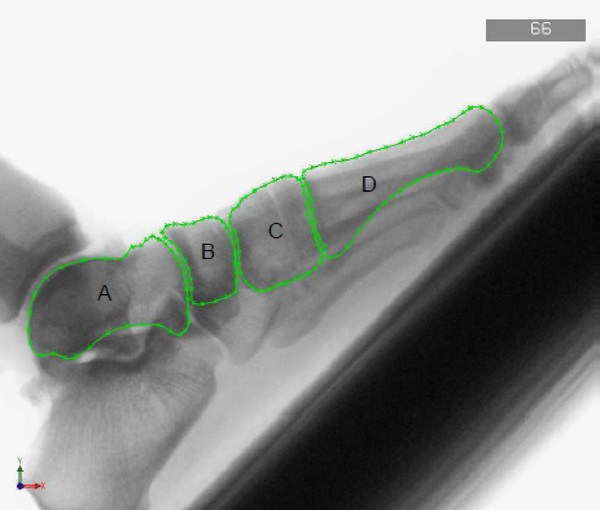
**Fluoroscopic image of the first ray imported into a CAD program with manually drawn outline of the bones, participant 1-left foot, Frame 66:A) talus, B) navicular bone, C) medial cuneiform, D) first metatarsal**.

A minimum of 20 points was manually distributed about the bone perimeter and perimeter approximation was performed with spline functions (Figure 2) using the *Spline *button within the sketcher of the CAD system. The area enclosed by the bone outlines was calculated as well as the principal axes of inertia and the coordinates of the center of gravity (*Tools → Section properties *function within the sketcher). These values were used to represent the position and the orientation of the bone projection within the image coordinate system.

Since the shape of the bones differed only very little from one frame to the next, the bone outlines were digitized in one frame and copied to the next frame for manual positioning and alignment.

**Figure 3:**
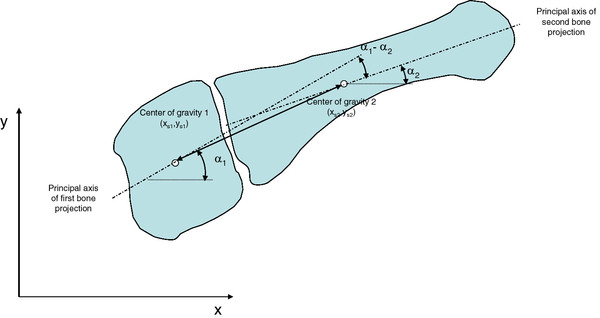
**Determination of the relative movement between two foot bones from the difference angle of the principal axes of inertia**.

After determination of the center of gravity and direction of the principal axes of inertia of the bone projections, the calculation of the dimensionless time, averaging and calculation of standard deviation was performed by spreadsheet software (Microsoft Excel). The distances between the centers of gravity and the angular differences between the principal axes of inertia were determined (Figure 3).

**Figure 4:**
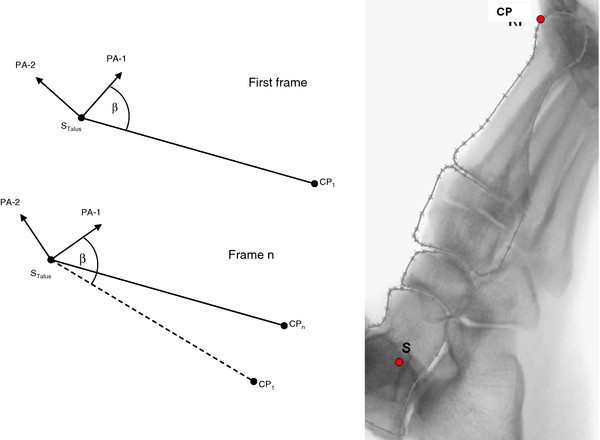
**Determination of the displacement of a control point (CP) as a measure for first ray mobility defined in a coordinate system fixed to the principal axes (PA) of inertia fixed to the talus**.

For the calculation of the dorsi-/plantar flexion, a control point (CP) was defined at the dorsal side of the outline of the head of the first metatarsal (MT-I) (Figure 4). The control point and its coordinates were manually selected using the CAD system. As a measure for first ray flexion, the displacement of the control point CP_1 _to CP_n _in a coordinate system fixed to the talus and defined by the principal axes of the talus was determined.

The foot roll-over process occured at different speeds among the subjects. For an easier comparison and for a unique time scale, a dimensionless time τ was defined:

τ = 0: heel strike

τ = 1: heel rise

τ = t/t_0_

t_0_: time between the heel strike and heel rise.

Before heel strike and after heel rise the images became more and more blurred since, with heel rise, rotation of the foot began. The center of rotation was located near the heads of the metatarsals. The talus having the highest distance from this pivot point had the highest velocity and consequently was the most blurred. Some of the images, which were recorded after heel rise, could be evaluated, since not all of these were excessively blurred and thus allowed analyses. These sequences were included in the evaluation because they show the first ray flexion during the gait phase without heel-ground contact. However, the highest first ray flexion was obtained around the time t_0_.

The different foot roll-over speed of the participants also influenced the quality of the images. Furthermore, in a few trials some relevant foot bones were outside of the images borders. Three image sets could not be evaluated for this reason. Another problem arose from the other leg swinging forward in the natural gait movement leading to a relevant overlay of both feet during registration. However, in the majority of image data sets, this overlay was obtained only on a very low number of images, which could be excluded from evaluation without additional inaccuracies.

To test the reproducibility of the contour detection and evaluation method, a randomly selected image was evaluated independently by another evaluation person and the results were compared. The maximum differences were found to be 0.19% with respect to the center of gravity coordinates and 1.42% with respect to the principal axes rotations.

Some image data sets did not show the heel or the forefoot fully within the image borders. Finally, some image data sets had to be excluded due too high blurriness because of very fast foot motion. Thus, from the 44 feet 27 could be evaluated (15 feet of healthy volunteers and 12 of patients), each of these with at least 10 digitized frames.

### Statistics

The significance of the difference of all relevant measured magnitudes between the patients and the healthy volunteers was tested by the Mann-Whitney test (non-parametric). The significance level was set to p < 0.05).

## Results

### First ray flexion values

**Figure 5:**
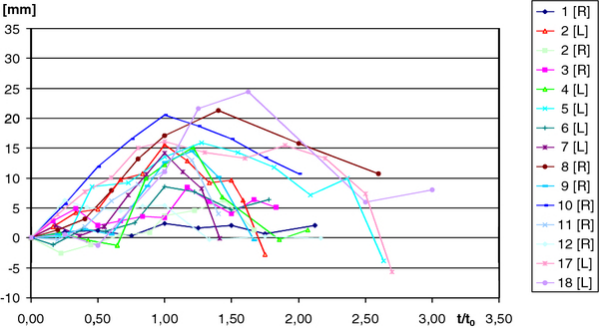
**First ray mobility in the healthy volunteers recorded over dimensionless time**.

The first ray flexion data of the healthy volunteers are displayed in Figure 5. After heel strike, the flexion of the first ray increased to a maximum value shortly after heel rise. The average of the maximum dorsal flexion was 13.63 (SD 6.14) mm. The plantar motion components were excluded from the calculation. The curves reached their maximum at an average time of τ = 1.11 (SD 0.31).

**Figure 6:**
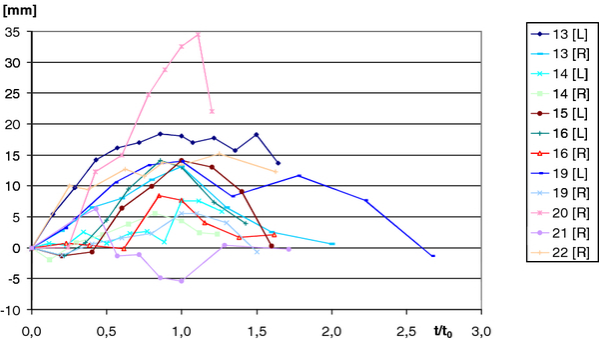
**First ray mobility in the patients recorded over dimensionless time**.

Figure 6 shows the first ray flexion of the patients. The average of the maximum dorsal flexion angle was 13.06 (SD 8.01) mm. The maximum values occurred on average with a time of τ = 0.94 (SD 0.21) (Table 1). The average dorsiflexion angle in the healthy volunteers was 5.27° (SD 2.34), and 5.56° (SD 3.37) in the patients.

**Table 1:** Maximum values of first ray mobility and the relative time of their occurrence

Subjects	Average [mm]	SD [mm]	t/t_0_	SD
healthy volunteers	13.63	6.14	**1.11**	0.31

Hallux valgus patients	13.06	8.01	**0.94**	0.21

(boldface-significant *p *< 0.05)

Only the difference in time of maximum first ray flexion occurrence was found to be significant.

### Relative rotations of foot bones

The results of the relative rotations of the bones in the sagittal plane (Table 2) did not show significant differences between the groups of the healthy volunteers and the patients. The average maximum values of the healthy volunteers were even slightly higher than the values of the patients. The highest difference amounted to 0.48° in the talonavicular articulation.

**Table 2:** Average values of maximum rotations of the bones to each other in the sagittal plane

articulation	healthy volunteers		hallux valgus patients	
	**Average [°]**	**t/t_0_**	**Average [°]**	**t/t_0_**

MT1/OCm	2.58	1.19	2.61	0.86

OCm/ONav	5.95	1.42	5.63	1.03

ONav/Talus	5.78	1.11	4.83	0.94

(MT1-first metatarsal, OCm-medial cuneiform, ONav-navicular bone)

The relative rotations in the naviculo-medial cuneiform articulation and in the talonavicular articulation were similar with values between 5.1° and 5.9°. In contrast, the maximum relative rotations between the first metatarsal and the medial cuneiform were approximately half of these values. The changes of rotations were not found to be significant.

### Relative translations of foot bones

The relative translations of foot bones to each other varied between 0.78 mm and 1.65 mm. With respect to the talus they reached 2.17 mm. These results were not found to be significant.

## Discussion

The foot is often described in current foot models by substantially fewer segments than the actually existing anatomic structures [7,14,19]. However, for the determination of instabilities and their specific anatomical localization occurring during the gait cycle, a more detailed analysis seems to be necessary in view of the fact that clinicians claim that instability in hallux valgus is mostly confined within the first tarso-metatarsal joint which indicates surgery in the form of an arthrodesis of this joint [4-6].

The first ray of the foot has an important function with respect to load transfer and stability [2]. The relevance of instability of the first ray in hallux vagus deformity has been a matter of discussion for decades. The particular pathogenesis of hallux valgus formation and deformity progression has been attributed to first ray instability with variable percentage [4-6,9,11].

To date, the diagnosis of a hypomobile or hypermobile first ray has mostly been performed by clinical assessment with a high degree of variance [5,20] or by static measurement methods at a limited dorsiflexion force of 55 N [9,10,12]. Consequently, as a result of these approaches the normal mobility of the first ray had been defined between 4 and 8 mm. Standard weight-bearing radiographs only add indirect and mostly inconstant static signs of medial cuneiform-first metatarsal instability (plantar joint opening, localized osteoarthritis or widened first intermetatarsal angle) [5,6,8].

Up to now, dynamic measurements of forefoot kinematics were mostly performed with opto-electronical methods. The values of the forefoot dorsal flexion in relation to the hindfoot in normals were found to be within the range of 3.0-6.2° [7,14,19,21,22].

Dynamic standard fluoroscopic analysis has been employed in a variety of in-vivo biomechanical evaluations, e.g. normal and anterior cruciate-deficient knee joint kinematic studies or kinematic studies following various types of knee joint arthroplasty [23-25]. In most application modes additional data acquisition such as CT scanning of the knee was necessary as a prerequisite for the generation of a mathematical 3-D model utilizing an iterative model-fitting approach. Data from conventional dynamic fluoroscopy itself can not readily be taken for direct measurements due to a substantial amount of magnification and distortion depending on the distance of the corresponding image point from the focus. The novel digital fluoroscopic acquisition tool used in this study allowed a distortion-free and detailed analysis of sagittal motion at the anatomic joints of the medial ray. Such detailed in-vivo analyses have not been possible previously. Of course, this novel method has several limitations and potential errors. The errors arising from the described evaluation method can to be divided into three categories:

(i) errors due to the spatial and temporal resolution of the imaging system,

(ii) errors due to two-dimensional imaging and evaluation,

(iii) errors due to manual positioning and digitalisation of the image.

These errors were estimated in more detail. The 25 frames/s imaging rates only allow the analysis of relatively slow walking speeds. In our chosen set-up, free walking was replaced by a single step analysis, as walking at a continuous speed would mean that the fluoroscope would also have to move. In principal, additional analysis of in-vivo kinematics in the horizontal plane during foot contact phase would be desirable [2,6] but could hardly be accomplished by the current technique due to an inevitable mechanical interference of the walking person with the fluoroscopic device turned to a vertical or oblique position.

The main source of errors was expected from (iii). The outline of bone was manually drawn into an image where all contours were well visible. However, some images, in particular during the last phase of roll-over process, were blurry so that errors arose from the transfer of the contours onto the subsequent images. Further, the determination of the medial foot column mobility depended on the correct determination of the talar contour. An angular deviation of 1° of malpositioning of the talar contour would change the first ray mobility result by about 2.5 mm. The errors from the other sources (i) and (ii) were considered to have only a minor influence on the results. Moreover, the errors (ii) and (iii) were minimized as much as possible by copying the bone contours from images where these contours could be well defined to the more blurry images.

A comparison of our results was made with data from the static analyses, with data obtained by the classic marker technique and with data from kinematic analyses by camera. Our data indicate a substantially higher first ray mobility than described in the literature for static measurements [9,10,12,13]. Taking into account the substantially higher loading during single-leg full weight-bearing as in our experiments, the limited loading during static measurements and the difference between static and dynamic values [7] this does not appear to be surprising. Between the groups of the healthy volunteers and the patients there were, however, only statistically non-significant differences with 13.6 mm and 13.1 mm, respectively.

More recent studies use an advanced combined 2D-3D model-image registration technique for foot kinematics in healthy subjects [26-29], patients with hallux valgus [30], hallux rigidus [22], flat-arched feet [31] and for subjects with ankle arthroplasty [32]. These studies report 15° of plantarflexion to 20° dorsiflexion with the healthy subjects with and without weight-bearing activities. The calculated angular values of forefoot dorsiflexion for both our two groups of subjects, nevertheless, were comparable to literature data of opto-electronical measurements ranging between 0.7 and 9.3° [7,14,19,21,22,27,33]. Furthermore, angular measurements are independent of linear measures, such as the individual foot length, and seem to be generally preferable compared to mere distance measures. With 5.3° and 5.6° only statistically non-significant differences between the groups of the healthy volunteers and the patients were found in our study. Present 3D multisegment foot models have been shown to have a very high reliability index for the sagittal plane kinematics. Moreover, they also yield data for the motion within the coronal and horizontal planes. However the adequate marker placement, soft tissue artifacts stereophotogrammetric-based marker position tracking and the basic assumptions of the corresponding foot model do have an influence on the calculations of the corresponding joint rotations [22,27,29,30].

The relative rotational movements in the sagittal plane in our study did not show distinguishable differences between both groups. Still, it is noticeable that, in contrast to clinical assumptions [5,8], an increased mobility at the first metatarsal-medial cuneiform articulation was not seen in either of our two groups. Compared with the navicular-medial cuneiform articulation and the talo-navicular joint even the smallest rotations were found at the first metatarsal-medial cuneiform articulation. This agrees well with data from in-vitro experiments [15,16] and reports from a limited number of invasive in vivo assessment of mid- and forefoot motion during walking [18] or slow running [28].

The groups of the healthy volunteers and the patients differed significantly in the time-point of occurrence of the maximum values of the first ray flexion and the relative rotations of the bones to each other. The motion diagrams within the group of patients reached their maxima with heel rise. In contrast, the motion diagrams of the healthy volunteer group reached their maximum values significantly later which might at least point to increased medial ray flexibility in the patient group despite a comparable total range of motion in both groups.

The translational relative motions between the foot bones are considered to be in the order of magnitude of the measurement precision. With very limited values between 1 and 2 mm a characteristic curve form could not be recognized.

The values of the standard deviation for the first ray flexion and the relative rotational motion of the bones were relatively high within both of our groups of test subjects. This indicates high inter-individual variations within the groups. Maximum rotational motions could be found mainly in the navicular-medial cuneiform articulation and in the talo-navicular articulation which has also been reported by invasive measurements of rear-, mid- and forefoot motion [18,28].

## Conclusions

The novel digital fluoroscopic-based method developed and described here can be used to analyze in vivo the rotational and the translational movements of the first metatarsal the medial cuneiform bone, the navicular bone and the talus within their articulations of the medial foot column. The procedure serves as a stand-alone technique to assess (with a resolution of 0.245 mm/pixel) the mobility of the individual bones making up the first ray in the sagittal plane and the relative movements of the single bones during weight-bearing. Such an assessment of individual bone mobility and relative movement of the single bones during weight bearing has not previously been possible by any of the other currently available non-invasive measurement techniques. This procedure may further aid to elucidate the biomechanics of the healthy and the diseased forefoot and mid-foot during gait and may be applied prospectively in conjunction with conventional opto-electronical devices.

## Competing interests

The authors declare that they have no competing interests.

## Authors' contributions

HM developed the method and carried out the results interpretation and statistics and drafted the manuscript. UB carried out the fluoroscopy image evaluation and the results calculation. AD participated in the patient selection, the study management and conduction. VZ participated in the study design and put the excellent experimental facilities of his institute to our disposal. KPS participated in the study design and in the biomechanical evaluation of the study data. TM conceived of the study, and participated in its design and coordination and helped to draft the manuscript. Moreover, HM, AD and TM took part in the study as volunteers. All authors read and approved the final manuscript.

## Pre-publication history

The pre-publication history for this paper can be accessed here:

http://www.biomedcentral.com/1471-2474/13/14/prepub

## References

[B1] Glasoe WM Nuckley DJ Ludewig PM Hallux valgus and the first metatarsal arch segment: a theoretical biomechanical perspective Phys Ther 2010 90 110 120 10.2522/ptj.20080298 19926679

[B2] Wukich DK Donley BG Sferry JJ Hypermobility of the first tarsometatarsal joint Foot Ankle Clin N Am 2005 10 157 166 10.1016/j.fcl.2004.11.004 15831264

[B3] Roukis TS Landsman AS Hypermobility of the first ray: a critical review of the literature J Foot Ankle Surg 2003 42 377 390 10.1053/j.jfas.2003.09.010 14688782

[B4] Faber FW Kleinrensink GJ Mulder PG Verhaar JA Mobility of the first tarsometarsal joint in hallux valgus patients: a radiographic analysis Foot Ankle Int 2001 22 965 969 11783922 10.1177/107110070102201207

[B5] Coughlin MJ Jones CP Hallux valgus and first ray mobility. A prospective study J Bone Joint Surg Am 2007 89 1887 1898 10.2106/JBJS.F.01139 17768183

[B6] Klaue K Hallux valgus and hypermobility of the first ray-causal treatment using tarso-metatarsal reorientation arthrodeses Ther Umsch 1991 48 817 823 1805443

[B7] Allen MK Cuddeford TJ Glasoe WM DeKam LM Lee PJ Wagner KJ Yack HJ Relationship between static mobility of the first ray and first ray, midfoot, and hindfoot motion during gait Foot Ankle Int 2004 25 391 396 15215023 10.1177/107110070402500605

[B8] Glasoe WM Coughlin MJ A critical analysis of Dudley Mortons concept of disordered foot function J Foot Ankle Surg 2006 45 147 155 10.1053/j.jfas.2006.02.008 16651193

[B9] Glasoe WM Yack HJ Saltzman CL Anatomy and biomechanics of first ray Phys Ther 1999 79 854 859 10479786

[B10] Glasoe WM Allen MK Saltzman CL First ray dorsal mobility in relation to hallux valgus deformity and first intermetatarsal angle Foot Ankle Int 2001 22 98 101 11249233 10.1177/107110070102200203

[B11] Grebing BR Coughlin MJ The effect of ankle position on the exam for first ray mobility Foot Ankle Int 2004 25 467 475 15319104 10.1177/107110070402500705

[B12] Klaue K Hansen ST Masquelet AC Clinical, quantitative assessment of first tarsometatarsal mobility in the sagittal plane and its relation to hallux deformity Foot Ankle Int 1994 15 9 13 7981800 10.1177/107110079401500103

[B13] Glasoe WM Grebing BR Beck S Coughlin MJ Saltzman CL A comparison of device measures of dorsal first ray mobility Foot Ankle Int 2005 26 957 961 16309611 10.1177/107110070502601111

[B14] Wolf P List R Ukelo T Maiwald C Stacoff A Day-to-day consistency of lower extremity kinematics during walking and running J Appl Biomech 2009 25 369 376 20095458 10.1123/jab.25.4.369

[B15] Ouzounian TJ Shereff MJ In vitro determination of midfoot motion Foot Ankle 1989 10 140 146 2613125 10.1177/107110078901000305

[B16] Nester CJ Liu AM Ward E Howard D Cocheba J Derrick T Patterson P In vitro study of foot kinematics using a dynamic walking cadaver model J Biomech 2007 40 1927 1937 10.1016/j.jbiomech.2006.09.008 17081548

[B17] Nester CJ Jones RK Liu A Howard D Lundberg A Arndt A Lundgren P Stacoff A Wolf P Foot kinematics during walking measured using bone surface markers J Biomech 2007 40 3412 3423 10.1016/j.jbiomech.2007.05.019 17631298

[B18] Lundgren P Nester C Liu A Arndt A Jones R Stacoff A Wolf P Lundberg A Invasive in vivo measurement of rear-, mid-, and forefoot motion during walking Gait Posture 2008 28 93 100 10.1016/j.gaitpost.2007.10.009 18096389

[B19] Carson MC Harrington ME Thompson N O'Connor JJ Theologis TN Kinematic analysis of multi-segment foot model for research and clinical applications: a repeatability analysis J Biomech 2001 34 1299 1307 10.1016/S0021-9290(01)00101-4 11522309

[B20] Cornwall MW Fishco WD McPoil TG Lane CR O'Donnell D Hunt L Reliability and validity of clinically assessing first-ray mobility of the foot J Am Podiatric Med Ass 2004 94 470 476 10.7547/0940470 15377723

[B21] Steiger C Day-to-day variability of orthopaedically relevant kinematic parameters in the foot 2008 ETH Zurich: Master thesis (english abstract)

[B22] Canseco K Long J Marks R Khazzam M Harris G Quantitative characterization of gait kinematics in patients with hallux rigidus using the Milwaukee foot model J Orthop Res 2008 26 419 427 10.1002/jor.20506 17972321

[B23] Dennis DA Mahfouz MR Komistek RD Hoff W In vivo determination of normal and anterior cruciate ligament-deficient knee kinematics J Biomech 2005 38 241 253 10.1016/j.jbiomech.2004.02.042 15598450

[B24] Komistek RD Dennis DA Mahfouz M In vivo fluoroscopic analysis of the normal human knee Clin Orthop 2003 410 69 81 12771818 10.1097/01.blo.0000062384.79828.3b

[B25] Komistek RD Kane TR Mahfouz M Ochoa JA Dennis DA Knee mechanics: a review of past and present techniques to determine in vivo loads J Biomech 2005 38 215 228 10.1016/j.jbiomech.2004.02.041 15598448

[B26] Yamaguchi S Sasho T Kato H Kuroyanagi Y Banks SA Ankle and subtalar kinematics during dorsiflexion-plantarflexion activities Foot Ankle Int 2009 30 4 361 366 10.3113/FAI.2009.0361 19356362

[B27] Leardini A Benedetti MG Berti L Bettinelli D Nativo R Giannini S Rear-foot, mid-foot and forefoot motion during the stance phase of gait Gait Posture 2007 25 453 462 10.1016/j.gaitpost.2006.05.017 16965916

[B28] Arndt A Wolf P Liu A Nester C Stacoff A Jones R Lundgren P Lundberg A Intrinsic foot kinematics measured in vivo during the stance phase of slow running J Biomech 2007 40 2672 2678 10.1016/j.jbiomech.2006.12.009 17368465

[B29] Deschamps K Staes F Roosen P Nobels F Desloovere K Bruyninckx H Matricali GA Body of evidence supporting the clinical use of 3D multisegment foot models: a systematic review Gait Posture 2011 33 338 349 10.1016/j.gaitpost.2010.12.018 21251834

[B30] Deschamps K Birch I Desloovere K Matricali GA The impact of hallux valgus on foot kinematics: a cross-sectional comparative study Gait Posture 2010 32 102 106 10.1016/j.gaitpost.2010.03.017 20451392

[B31] Levinger P Murley GS Barton CJ Cotchett MP McSweeney SR Menz HB A comparison of foot kinematics in people with normal and flat-arched feet using the Oxford foot model Gait Posture 2010 32 519 523 10.1016/j.gaitpost.2010.07.013 20696579

[B32] Yamaguchi S Tanaka Y Kosugi S Takakura Y Sasho T Banks SA In vivo kinematics of two-component total ankle arthroplasty during non-weightbearing and weightbearing dorsiflexion/plantarflexion J Biomech 2011 44 6 995 1000 10.1016/j.jbiomech.2011.02.078 21392769

[B33] Leardini A Benedetti MG Catani F Simoncini L Giannini S An anatomically based protocol for the description of foot segment kinematics during gait Clin Biomech 1999 14 528 36 10.1016/S0268-0033(99)00008-X 10521637

